# Prognostic and predictive value of circulating tumor cells and CXCR4 expression as biomarkers for a CXCR4 peptide antagonist in combination with carboplatin-etoposide in small cell lung cancer: exploratory analysis of a phase II study

**DOI:** 10.1007/s10637-017-0446-z

**Published:** 2017-03-15

**Authors:** Ravi Salgia, R. Waide Weaver, Michael McCleod, John R. Stille, S. Betty Yan, Stephanie Roberson, John Polzer, Amy Flynt, Eyas Raddad, Victoria L. Peek, Sameera R. Wijayawardana, Suzane L. Um, Steve Gross, Mark C. Connelly, Carrie Morano, Madeline Repollet, Renouard Sanders, Kurt Baeten, David D’Haese, David R. Spigel

**Affiliations:** 1 City of Hope Comprehensive Cancer Center, 1500 E. Duarte Road, Duarte, CA 91010-3000 USA; 2 Florida Cancer Specialists, St. Petersburg, FL USA; 3 Florida Cancer Specialists, Fort Myers, FL USA; 4 The Chorus Group, Eli Lilly and Company, Indianapolis, IN USA; 5 Lilly Research Laboratories, Eli Lilly and Company, Indianapolis, IN USA; 6 PharPoint Research Inc., Durham, NC USA; 7 Janssen Diagnostics, Johnson and Johnson Company, Raritan, NJ USA; 8 Janssen Diagnostics, Janssen Pharmaceutica, Beerse, Belgium; 9 Sarah Cannon Research Institute, Nashville, TN USA

**Keywords:** LY2510924, CXCR4 expression, Circulating tumor cells, Small cell lung cancer, Carboplatin-etoposide

## Abstract

*Background* Circulating tumor cells (CTCs) and chemokine (C-X-C motif) receptor 4 (CXCR4) expression in CTCs and tumor tissue were evaluated as prognostic or predictive markers of CXCR4 peptide antagonist LY2510924 plus carboplatin-etoposide (CE) versus CE in extensive-stage disease small cell lung cancer (ED-SCLC). *Methods* This exploratory analysis of a phase II study evaluated CXCR4 expression in baseline tumor tissue and peripheral blood CTCs and in post-treatment CTCs. Optimum cutoff values were determined for CTC counts and CXCR4 expression in tumors and CTCs as predictors of survival outcome. Kaplan-Meier estimates and hazard ratios were used to determine biomarker prognostic and predictive values. *Results* There was weak positive correlation at baseline between CXCR4 expression in tumor tissue and CTCs. Optimum cutoff values were H-score ≥ 210 for CXCR4^+^ tumor, ≥7% CTCs with CXCR4 expression (CXCR4^+^ CTCs), and ≥6 CTCs/7.5 mL blood. Baseline H-score for CXCR4^+^ tumor was not prognostic of progression-free survival (PFS) or overall survival (OS). Baseline CXCR4^+^ CTCs ≥7% was prognostic of shorter PFS. CTCs ≥6 at baseline and cycle 2, day 1 were prognostic of shorter PFS and OS. None of the biomarkers at their respective optimum cutoffs was predictive of treatment response of LY2510924 plus CE versus CE. *Conclusions* In patients with ED-SCLC, baseline CXCR4 expression in tumor tissue was not prognostic of survival or predictive of LY2510924 treatment response. Baseline CXCR4^+^ CTCs ≥7% was prognostic of shorter PFS. CTC count ≥6 at baseline and after 1 cycle of treatment were prognostic of shorter PFS and OS.

**Electronic supplementary material:**

The online version of this article (doi:10.1007/s10637-017-0446-z) contains supplementary material, which is available to authorized users.

## Introduction

Small cell lung cancer (SCLC) accounts for approximately 10–15% of all lung cancer cases and is characterized by aggressive growth, early development of metastases, high mortality, and initial response to chemotherapy followed by relapse [1]. Approximately 60–70% of patients with SCLC have extensive-stage disease (ED) [2]. The 5-year survival rate for patients with SCLC is only 7% [3]. Patients with ED-SCLC have a median survival of 7–11 months with currently available therapy, and long-term disease-free survival is rare [4]. Despite development of targeted therapies for non-small cell lung cancer (NSCLC), there have been few advances in treatment options for patients with SCLC.

To date, no predictive validated biomarker is available for SCLC outcomes. A meta-analysis showed that elevated chemokine (C-X-C motif) receptor 4 (CXCR4) expression correlated with shorter overall survival (OS) in patients with NSCLC and suggested a poor prognostic outcome of this disease [5]. The value of CXCR4 expression as a prognostic marker of SCLC is not known. A lack of preclinical models or access to patient tissues samples, as surgery is rarely used to treat SCLC, yields few tumor specimens for research. Tumor biopsies of SCLC are difficult to obtain, frequently with low tumor purity, and the tissue is often necrotic [6]. Repeat biopsies are even more problematic, which compounds the challenges.

Collection of circulating tumor cells (CTCs) permits evaluation of biomarkers such as CXCR4 expression in patients with SCLC, as CTCs are present in high numbers in the peripheral blood of patients with SCLC [7–9]. This facilitates using CTCs as a liquid biopsy to examine genetic or phenotypic markers in patients with SCLC, and provides an opportunity for repeated sampling during the course of treatment. In addition to evaluating CXCR4 expression in CTCs, recent reports have suggested that CTCs alone may be a useful prognostic or predictive factor for survival in patients with SCLC, both at baseline and following 1 or 2 cycles of chemotherapy [10–12]. Cheng et al. [13] reported that, following a second cycle of treatment, CTCs and change in CTC counts from baseline were independent indicators for both progression-free survival (PFS) and OS in patients with ED-SCLC. In a pilot study analyzing CTCs as a biomarker of chemotherapy response and relapse, higher baseline CTCs in treatment-naïve patients with ED-SCLC and the percentage of decrease in post-treatment CTCs were associated with decreased survival [14].

CXCR4 is implicated in tumor cell motility, survival, and growth and is often overexpressed in SCLC tumors and SCLC cell lines [15–17]. CXCR4 activation in SCLC has been shown to induce migration and invasion into the extracellular matrix; marrow stromal cells or extracellular matrix proteins may protect SCLC cells from chemotherapy-induced apoptosis [15, 18, 19], suggesting a role for CXCR4 in chemotherapy resistance. Stromal cell-derived factor-1 (SDF-1, C-X-C motif chemokine 12) is an important ligand of CXCR4 [20].

A phase II study of LY2510924, a novel cyclic peptide that blocks the binding of the ligand SDF-1 (CXCL12) to CXCR4 [16, 21], was conducted in patients with ED-SCLC who received LY2510924 plus carboplatin-etoposide (CE) versus CE alone (NCT01439568) [22]. There was no difference in median PFS, the primary efficacy endpoint, between the treatment arms in this study [23], which was approximately 5.9 months in both arms. In exploratory analyses of patients with ED-SCLC and high CXCR4-expressing tumors at baseline (H-score ≥ 210), the hazard ratio (HR) for PFS between the treatment arms was 0.787 (95% confidence interval [CI]: 0.211, 2.933) [24]. Thus, CXCR4 overexpression in SCLC tumors does not appear to be a predictive biomarker for treatment response to LY2510924 plus CE versus CE alone. Here, the aim of our post-hoc exploratory analyses was to evaluate the potential prognostic value of CTC counts and CXCR4 expression in CTCs and in tumor tissue in the overall study population, and to evaluate potential predictive value of these biomarkers for treatment with LY2510924 plus CE versus CE alone. In addition, we evaluated the correlation of baseline CXCR4 expression in tumor tissue and in CTCs from patients in this phase II study.

## Methods

### Patients and treatments

In a phase II study [22], patients with ED-SCLC were randomized 1:1 to receive CE versus LY2510924 plus CE. Carboplatin (area under the curve [AUC] 5) and etoposide (100 mg/m^2^) were administered intravenously on day 1 and days 1–3, respectively, for up to six 21-day cycles. LY2510924 (20 mg) was administered subcutaneously daily on days 1–7 for up to six 21-day cycles. In accordance with the Declaration of Helsinki, written informed consent was obtained from all enrolled patients before initiating any study procedure. The study protocol was approved by an ethics committee and conformed to principles of Good Clinical Practice.

### Isolation and enumeration of CTCs and detection of CXCR4 expression

Blood from patients with ED-SCLC was collected into a CellSave preservative tube (Janssen Diagnostics, Raritan, NJ) at baseline (day 1 of cycle 1), day 7 of cycle 1, day 1 of cycle 2, and at 30-day follow-up after the last dose of study drug. Circulating tumor cells were isolated and enumerated using the CXC CELLSEARCH® kit and CELLSEARCH® system according to the manufacturer’s instructions (Janssen Diagnostics LLC, Raritan, NJ) as previously described [25]. Details of the CTC assay for CXCR4 expression detection are provided in Supplementary Materials and Methods. After CTC enumeration of each sample, CXCR4 expression was determined by an operator and defined as CXCR4-positive or -negative by visual phenotyping and classification. Details of CXCR4 quantification are provided in the Supplementary Materials and Methods. Representative images of tumor cell lines expressing varied levels of CXCR4 and a CXCR4+ CTC sample from a patient with SCLC are shown in Supplementary Fig. S1a–g.

### Baseline CXCR4 expression in tumors by immunohistochemistry

CXCR4 expression analysis was performed on formalin-fixed, paraffin-embedded tumor tissue. Immunohistochemistry (IHC) staining for CXCR4 (rabbit anti-human CXCR4 monoclonal antibody, clone UMB2; Epitomics Inc., Burlingame, CA) was performed at Bostwick Laboratories (Uniondale, NY) on an intelliPATH FLX AutoStainer (Biocare Medical, Concord, CA) using heat-induced antigen retrieval and standard techniques with 3,3′-diaminobenzidine as chromogen and hematoxylin counterstain. Stained slides were reviewed by board-certified pathologists; an H-score (0–300) for CXCR4 expression was assigned to each sample. Immunostaining intensity was assessed by visual assessment: 0 (no staining), 1+ (low intensity), 2+ (intermediate intensity), and 3+ (strong intensity). H-scores were calculated from the percentage of cells (in increments of 10%) at different staining intensities, using the formula: 0 (% of 0 staining cells) + 1 x (% of 1+ staining cells) + 2 x (% of 2+ staining cells) + 3 x (% of 3+ staining cells).

### Statistical analysis methods

A 2-sided significance (alpha) level of 0.05 was used to identify variables that may be predictive of PFS at 6 months or OS at 11.5 months. All other statistical testing was 2-sided at alpha level of 0.10. These analyses were hypotheses-generating and no adjustments for multiple comparisons were made; results should be interpreted with caution due to the possibility of a false positive. All statistical analyses were performed using SAS® version 9.2.

Biomarkers of CTC counts and CXCR4 expression in tumors and CTCs were summarized for the overall study population and by treatment arm. Baseline values were compared between treatment arms using a Mann-Whitney test. The Pearson product moment correlation coefficient for baseline CXCR4 expression in tumor tissue versus CTCs was calculated. Receiver operating characteristic (ROC) curves, AUCs, and Wald 95% CIs were generated using PROC LOGISTIC to determine optimum biomarker cutoffs (i.e., values that optimize sensitivity plus specificity). PFS and OS were evaluated at 4 and 6 months by optimum biomarker cutoffs using Kaplan-Meier analysis and log-rank test using PROC LIFETEST, with a Cox proportional hazards regression analysis using PROC PHREG to determine the HR and corresponding 95% CI.

## Results

### Patient characteristics and biomarker levels

Patient characteristics and demographics were published previously [23]. Of the 94 patients randomized, 90 ED-SCLC patients received treatment with LY2510924 plus CE (*N* = 47) or CE (*N* = 43) and comprised the overall study population. The present exploratory analyses included data from the efficacy population of 89 patients (Table 1): LY2510924 plus CE (*N* = 47) or CE (*N* = 42); data from 1 patient in the CE arm were excluded due to sub-therapeutic dosing on cycle 1, day 1 (protocol violation). The CXCR4 detection assay in CTCs became available after the study had started, and 25 of 292 samples had CTC counts without CXCR4 expression evaluation. A total of 15 patient blood samples did not yield evaluable CTC data due to insufficient sample volume, interference substance detected in enriched CTCs, or incorrect sample handling.

**Table 1:** Baseline tumor CXCR4 expression by IHC and CTC counts and CXCR4 expression in CTCs (%CXCR4^+^ CTC) at baseline and post-treatment by treatment arm or overall study population

Biomarker	LY2510924 + CE (*N* = 47)	CE (*N* = 42)	Total (*N* = 89)
CXCR4^+^ Tumor Tissue			
Baseline			
Patients with evaluable results, *n*	36	33	69
Mean H-score (SD)	205.8 (80.4)	199.8 (85.2)	203.0 (82.2)
Median H-score (range)	200.0 (20, 300)	200.0 (5, 300)	200.0 (5, 300)
*P*-value*			0.808
CTC Count			
Baseline			
Patients with evaluable results, *n*	42	36	78
Mean (SD)	615.5 (1419.4)	1125.7 (3865.7)	851.0 (2816.2)
Median (range)	20.5 (0, 7153)	98.5 (0, 21,428)	51.0 (0, 21,428)
*P*-value*			0.262
Patients with CTC, *n/N* (%)	33/42 (78.6)	32/36 (88.9)	65/78 (83.3)
Cycle 1, day 7			
Patients with evaluable results, *n*	32	30	62
Mean (SD)	118.2 (461.3)	57.4 (156.1)	88.8 (347.3)
Median (range)	4.0 (0, 2624)	2.5 (0, 594)	2.5 (0, 2624)
Patients with CTC, *n/N* (%)	23/32 (71.9)	19/30 (63.3)	42/62 (67.7)
Cycle 2, day 1			
Patients with evaluable results, *n*	34	27	61
Mean (SD)	70.6 (209.3)	25.1 (79.9)	50.5 (165.5)
Median (range)	1.5 (0, 875)	0.0 (0, 406)	0.0 (0, 875)
Patients with CTC, *n/N* (%)	18/34 (52.9)	12/27 (44.4)	30/61 (49.2)
30-day follow-up			
Patients with evaluable results, *n*	29	25	54
Mean (SD)	128.5 (476.3)	5.2 (18.1)	71.4 (351.9)
Median (range)	0.0 (0, 2008)	0.0 (0, 87)	0.0 (0, 2008)
Patients with CTC, *n/N* (%)	14/29 (48.3)	9/25 (36.0)	23/54 (42.6)
%CXCR4^+^ CTCs			
Baseline			
Patients with evaluable results, *n*	37	33	70
Mean (SD)	21.0 (26.3)	26.6 (24.7)	23.6 (25.5)
Median (range)	10.9 (0.0, 100.0)	21.1 (0.0, 100.0)	17.8 (0.0, 100.0)
*P*-value*			0.182
Cycle 1, day 7			
Patients with evaluable results, *n*	29	27	56
Mean (SD)	0.1 (0.2)	9.2 (17.5)	4.4 (12.9)
Median (range)	0.0 (0.0, 1.0)	0.0 (0.0, 58.3)	0.0 (0.0, 58.3)
Cycle 2, day 1			
Patients with evaluable results, *n*	33	24	57
Mean (SD)	7.9 (15.0)	10.6 (19.7)	9.1 (17.0)
Median (range)	0.0 (0.0, 50.0)	0.0 (0.0, 66.6)	0.0 (0.0, 66.7)
30-day follow-up			
Patients with evaluable results, *n*	29	23	52
Mean (SD)	4.1 (13.2)	8.7 (28.8)	6.1 (21.4)
Median (range)	0.0 (0.0, 50.0)	0.0 (0.0, 100.0)	0.0 (0.0, 100.0)

*CE* Carboplatin-etoposide, *CTC* Circulating tumor cell, *CXCR4* Chemokine (C-X-C motif) receptor 4, *IHC* Immunohistochemistry, *N* number of patients, *n* Number of patients in a category, *SD* Standard deviation

*P-value from 2-sample, 2-sided Mann-Whitney of null hypothesis that baseline values were same for treatment arms

There were no statistical differences between treatment arms for baseline tumor CXCR4 expression, CXCR4 expression in CTCs, and CTC counts (Table 1). At baseline, 83% of the overall study population had ≥1 CTCs/7.5 mL blood with a mean of 851.0 and median of 51.0. The baseline %CXCR4^+^ CTCs mean was 23.6 with a median of 18.0. The CXCR4^+^ tumor tissue H-score mean was 203.0 with a median of 200.0. For the overall study population, baseline CXCR4 expression in tumor tissue positively correlated with baseline %CXCR4^+^ CTCs (*r* = 0.423, 95% CI = 0.174, 0.616; *P* = 0.001) (Supplementary Table S1; Supplementary Fig. S2a).

### Prognostic value of biomarker levels for PFS and OS

The phase II study demonstrated no difference in outcomes between the 2 treatment arms [23]; therefore, the overall study population data were combined to determine optimum cutoffs based on ROC for 6-month PFS (Supplementary Table S2; Supplementary Fig. S2b–f). These optimum cutoff values were: H-score ≥ 210 for CXCR4^+^ tumor tissue, CTCs ≥6/7.5 mL blood, and CXCR4^+^ CTCs ≥7%, and were evaluated by treatment and visit (Supplementary Table S3) or endpoint (Supplementary Table S4). The AUC values for these 3 optimum cutoffs with sensitivity, specificity, and positive and negative predictive values are shown in Supplementary Table S2. Baseline %CXCR4^+^ CTCs was the only biomarker with a lower limit of the CI for AUC >0.5 (AUC = 0.702; CI = 0.577, 0.828; *P* = 0.011) and a significant *P* < 0.05.

**Fig. 1:**
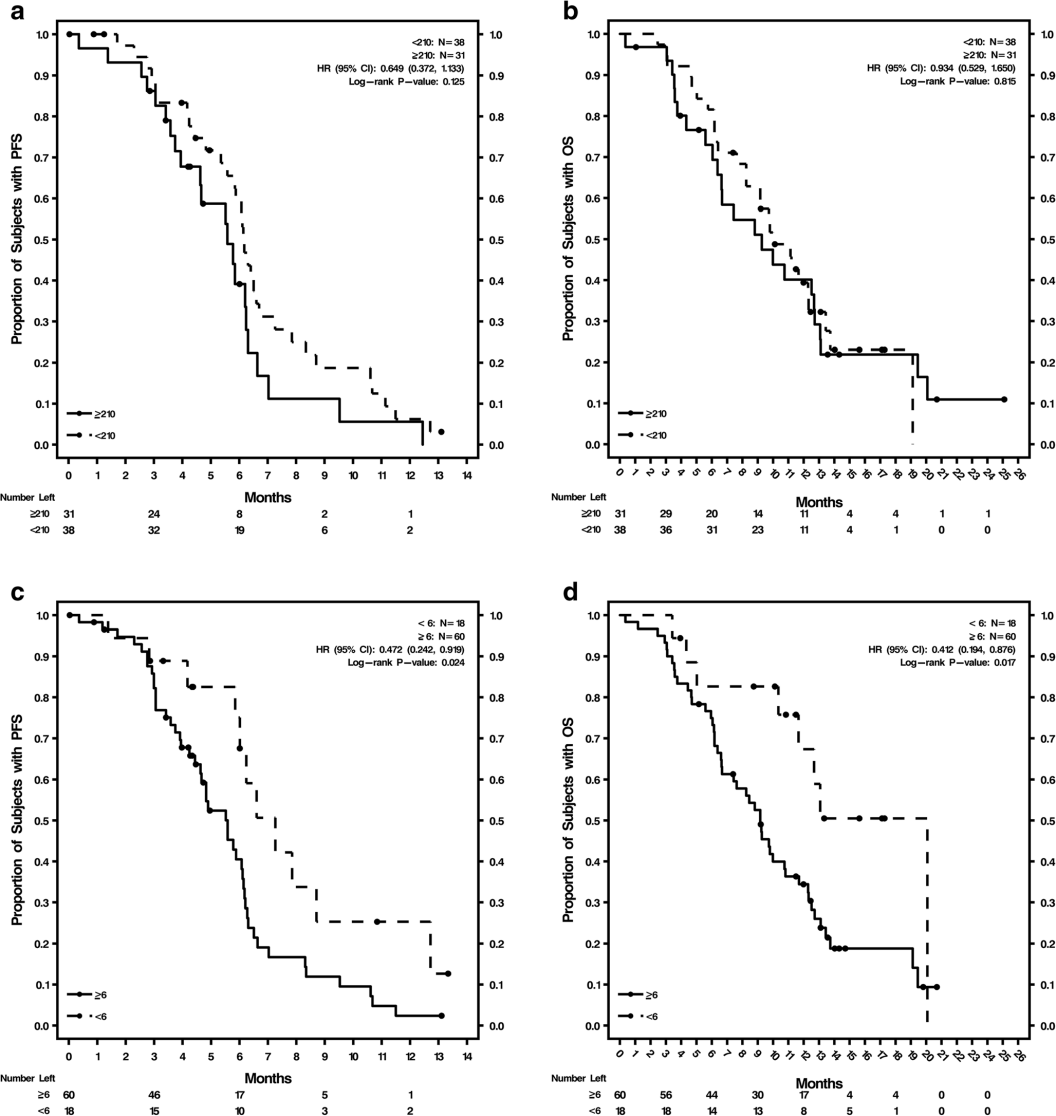

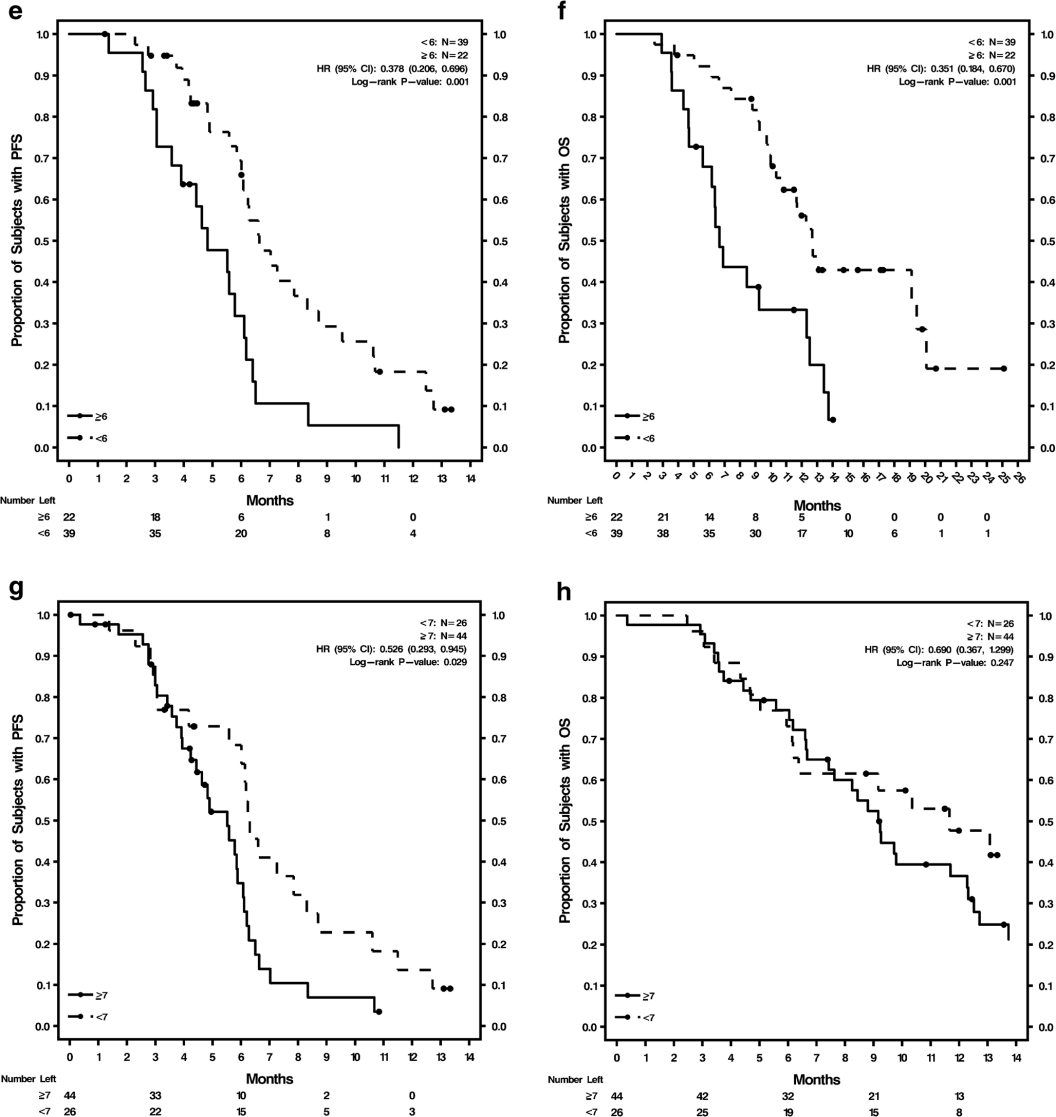

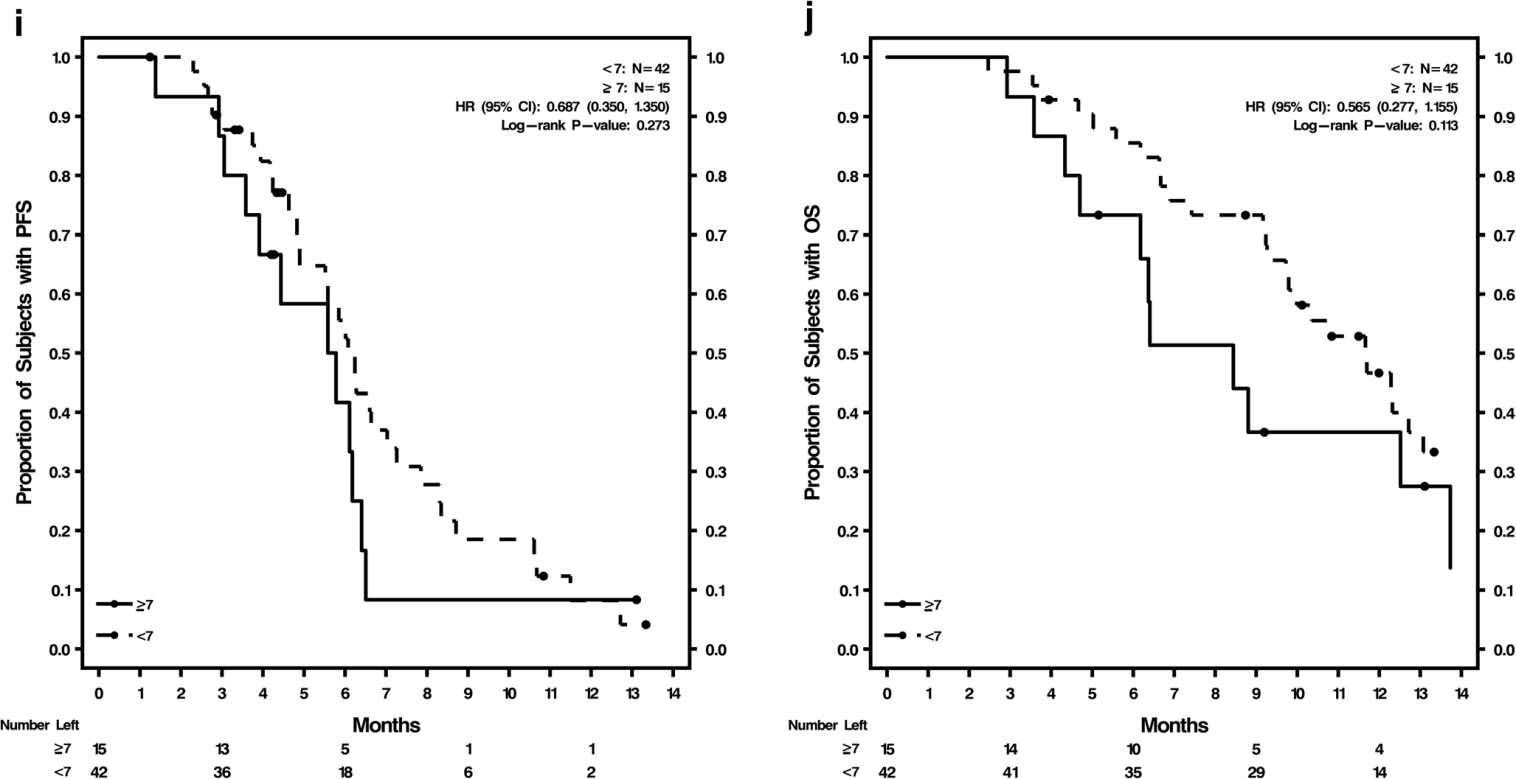
Kaplan-Meier curves for PFS and OS in the overall patient population by biomarker levels **a** Baseline CXCR4 expression in tumor tissue H-score ≥ 210 and <210 for PFS; **b** Baseline CXCR4 expression in tumor tissue H-score ≥ 210 and <210 for OS; **c** Baseline CTC counts ≥6 and <6 for PFS; **d** Baseline CTC counts ≥6 and <6 for OS; **e** Cycle 2, day 1 CTC counts ≥6 and <6 for PFS; **f** Cycle 2, day 1 CTC counts ≥6 and <6 for OS; **g** Baseline %CXCR4^+^ CTCs ≥7% and <7% for PFS; **h** Baseline %CXCR4^+^ CTCs ≥7% and <7% for OS; **i** Cycle 2, day 1 %CXCR4^+^ CTCs ≥7% and <7% for PFS; **j** Cycle 2, day 1 %CXCR4^+^ CTCs ≥7% and <7% for OS

Kaplan-Meier survival estimates and Cox regression analyses were done using optimum cutoffs of these 3 biomarkers, comparing patients above and below the cutoffs in the overall study population (Fig. 1a–j; Supplementary Table S5). The analyses were conducted using the cutoffs at baseline and at cycle 2, day 1 for both PFS and OS. Baseline tumor tissue CXCR4 expression was not prognostic of either PFS or OS in the overall study population. The Kaplan-Meier curves (Fig. 1a, b) were not well separated for H-scores of ≥210 versus <210. CTCs ≥6 were prognostic of shorter PFS and OS at baseline and at cycle 2, day 1: CTCs at baseline were significant indicators for PFS (*P* = 0.024) and OS (*P* = 0.017), and CTCs at cycle 2, day 1 were significant indicators for PFS (*P* = 0.001) and OS (*P* = 0.001). Kaplan-Meier curves for PFS and OS were well separated for CTCs ≥6 versus <6 at baseline (Fig. 1c, d) and cycle 2, day 1 (Fig. 1e, f). Baseline CXCR4^+^ CTCs ≥7% was prognostic of shorter PFS (*P* = 0.029) but not OS (Supplementary Table S5; Fig. 1g, h). CXCR4^+^ CTCs ≥7% at cycle 2, day 1 was not prognostic of either PFS or OS (Supplementary Table S5; Fig. 1i, j).

### Predictive value of biomarker levels for PFS and OS by treatment arm

**Fig. 2:**
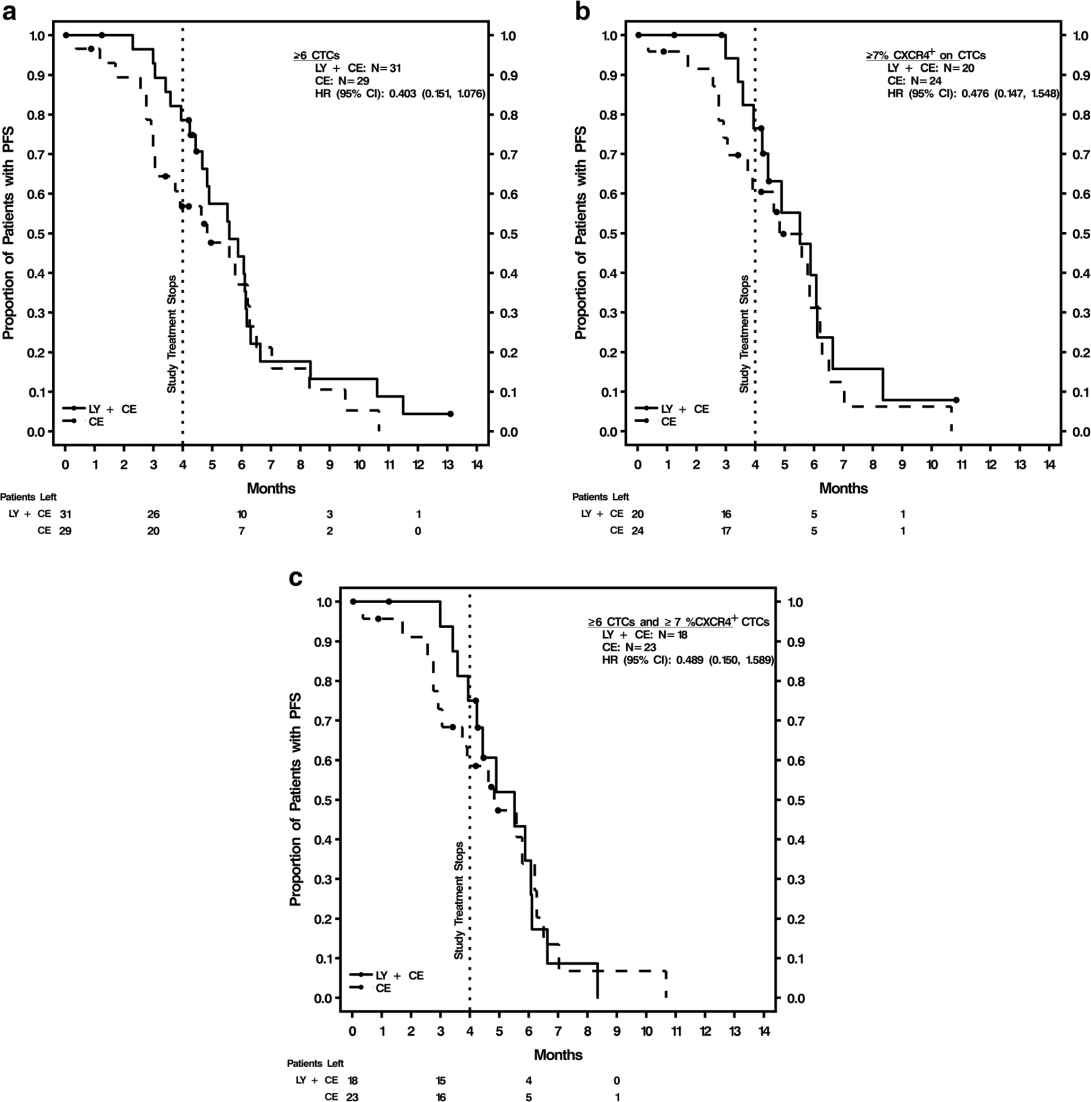
Kaplan-Meier curves for PFS by treatment arm and biomarker levels. **a** Baseline ≥6 CTCs; **b** Baseline ≥7% CXCR4^+^ CTCs; **c** Baseline ≥7% CXCR4^+^ CTCs and ≥6 CTCs. *CE* carboplatin-etoposide, *CI* confidence interval, *CTCs* circulating tumor cells, *CXCR4* chemokine (C-X-C motif) receptor 4, *HR* hazard ratio at 4 months (end of treatment), *LY* LY2510924, *N* number of patients, *OS* overall survival, *PFS* progression-free survival

The optimum cutoffs for the 3 biomarkers (H-score ≥ 210 for CXCR4^+^ tumor tissue, CTCs ≥6, and CXCR4^+^ CTCs ≥7%) were applied to survival analyses by treatment arm. Kaplan-Meier survival estimates (in months) and HR values for PFS and OS at 4 and 6 months are shown by treatment arm in Supplementary Table S4. All 3 biomarkers were analyzed at baseline, and CTCs and %CXCR4^+^ CTCs at cycle 2, day 1. None of the 3 biomarkers at their respective optimum cutoffs was predictive of treatment response. Representative Kaplan-Meier curves are shown in Fig. 2, including PFS for patients with baseline CTCs ≥6 and PFS for patients with baseline CXCR4^+^ CTCs ≥7%, by treatment arm (Fig. 2a, b). The Kaplan-Meier curves were separated in the first 4 months of treatment, but were no longer separated after approximately 4–5 months. Per study design, patients were on treatment for a maximum of six 21-day cycles, which was approximately 4 months. An analysis was conducted for patients with both baseline CTCs ≥6 and CXCR4^+^ CTCs ≥7%, considered a higher-risk group, to evaluate whether a combined elevation of these biomarkers may be predictive of treatment response as measured as PFS and OS. Kaplan-Meier survival curves and HR values show that this combination of elevated baseline biomarkers was not predictive of PFS (Table 2; Fig. 2c) or OS (data not shown).

**Table 2:** Predictive value of combined elevated baseline markers for PFS (4 months or 6 months) by treatment arm

	LY2510924 + CE (*N* = 18)	CE (*N* = 23)	Total (*N* = 41)
Baseline ≥7% CXCR4^+^ CTCs and ≥6 CTC count			
Kaplan-Meier estimate (mos)			
Mean (SD)	5.3 (0.4)	4.9 (0.6)	5.0 (0.4)
Median (95% CI)	5.5 (3.9, 6.1)	4.8 (2.9, 6.2)	4.9 (3.9, 6.1)
HR LY2510924 + CE vs CE (95% CI)			0.94 (0.46, 1.95)
*P*-value*			0.872
HR LY2510924 + CE vs CE (95% CI) 4 mos			0.49 (0.15, 1.59)
*P*-value through 4 mos*			0.223
HR LY2510924 + CE vs CE (95% CI) 6 mos			0.79 (0.34, 1.85)
*P*-value through 6 mos*			0.588

*CE* Carboplatin-etoposide, *CI* Confidence interval, *CTCs* Circulating tumor cell, *HR* Hazard ratio, *mos* Months

*P-value from a log-rank test

## Discussion

In a phase II study (NCT01439568 [22]) of LY2510924, a cyclic peptide that blocks the binding of the ligand SDF-1 (CXCL12) to CXCR4 [16, 21], there was no difference in median PFS for patients with ED-SCLC treated with LY2510924 plus CE and CE [23]. We conducted post-hoc exploratory analyses to evaluate the prognostic value of CTC counts and CXCR4 expression in both CTCs and tumor tissue in the overall study population, the predictive value of these biomarkers for treatment response to LY2510924 plus CE versus CE alone, and the correlation of CXCR4 expression in CTCs and tumors. These exploratory analyses were done on a limited dataset with no adjustments for multiplicity, and the results should be considered as hypotheses that need further testing. The proportion of patients (83%) in our study with ≥1 CTC/7.5 mL blood at baseline was similar to Normanno et al. [26]. The median CTC count at baseline in our study is comparable to reports in the literature for SCLC (Hou et al. [8], Huang et al. [14], and Normanno et al. [26]).

The CELLSEARCH system has been used to detect CTCs in various tumor types, including SCLC, making CTC counts or characterization a useful biomarker to establish cutoffs [9, 12, 14]. In the present analyses using CELLSEARCH, an optimum cutoff of ≥6 CTCs/7.5 mL blood at baseline and post-treatment (cycle 2, day 1) was prognostic of shorter PFS and OS. There were 77% and 36% of the patients in this study with baseline and cycle 2, day 1 CTC counts ≥6, respectively. Other studies have defined variable CTC cutoffs that demonstrated prognostic value for treatment outcomes: 50 CTCs/7.5 mL by Hou et al. [9], 8 CTCs/7.5 mL by Naito et al. [12], 2 CTCs/7.5 mL by both Hiltermann et al. [10] and Wu et al. [27], 5 CTCs/7.5 mL by Cheng et al. [28], and 282 CTCs/7.5 mL by Normanno et al. [26]. In our analyses, a cutoff of ≥6 CTCs was prognostic of both PFS and OS but was not predictive of 4- or 6-month PFS for treatment with LY2510924 plus CE versus CE. To our knowledge, this was the first analysis of CXCR4 expression in CTCs in SCLC, and a comparison of CXCR4 expression in tumor and CTCs (which may derive from the primary tumor or metastatic sites) showed a weak positive correlation. CXCR4 baseline overexpression in tumor (≥210 H-score) was not prognostic of shorter PFS or OS in patients with ED-SCLC. Baseline overexpression of CXCR4 in CTCs (≥7% CXCR4^+^ CTCs) was prognostic of shorter PFS, but not OS. Post-treatment (cycle 2, day 1) overexpression of CXCR4 in CTCs (≥7% CXCR4^+^ CTCs) was not prognostic of PFS or OS.

In both treatment arms, we observed median CTC counts and median %CXCR4+ CTCs decreases from baseline. Our data showed that if CTCs are ≥6 at cycle 2, day 1 it is a very strong prognostic biomarker of poor survival outcome (PFS and OS). Our data are consistent with several reports showing that when CTCs are decreased in response to chemotherapy in patients with SCLC, this can serve as a prognostic biomarker. Naito et al. [12] showed that patients with post-treatment CTCs ≥8 had worse outcomes versus CTCs <8 (*P* = 0.0096); patients with ≥8 CTCs at baseline also had a worse prognosis versus <8 CTCs at baseline (*P* = 0.0014). A study by Hou et al. [9] of patients with SCLC treated with first-line chemotherapy reported a decrease from pre-treatment CTC counts after 1 cycle of chemotherapy, which was an independent prognostic factor, and a higher number of CTCs were associated with shorter OS and PFS. Similarly, Cheng et al. [28] reported that in Chinese patients with ED-SCLC following 2 cycles of chemotherapy, ≥5 CTCs/7.5 mL decreases led to significantly shorter median OS versus <5 CTCs/7.5 mL blood (*P* < 0.0124); multivariate analyses showed CTCs was an independent prognostic marker for OS at baseline (*P* = 0.018) and after 2 cycles of chemotherapy (*P* = 0.0249). Overall, CTC counts appear to be a promising prognostic biomarker for ED-SCLC; collecting CTCs from peripheral blood as a liquid biopsy, amenable to repeat sampling, circumvents a need for invasive, inaccessible, solid biopsies in this patient population.

CXCR4 is overexpressed in a variety of human cancers including breast, lung, kidney, colon, ovarian, and brain [29]. The landscape of clinical development of anti-CXCR4 therapies is emerging, and, at present, the prognostic role of CXCR4 overexpression as a biomarker across different tumors is unclear. This overexpression can correlate with increased risk for recurrence and poor survival, depending on tumor type and outcomes studied. Minamiya et al. [30] reported that in patients with adenocarcinoma of the lung, higher levels of tumor cell CXCR4 expression are prognostic of better outcomes. In contrast, Li et al. [31] reported that CXCR4 co-expression with urokinase-type plasminogen activator receptor predicts worse prognosis of SCLC patients. In a meta-analysis, the expression of CXCR4 was an independent prognostic factor for lower survival and increased metastasis in SCLC [32]. In our analysis, despite a weak positive correlation between CXCR4^+^ tumor and CXCR4^+^ CTCs, CXCR4 overexpression in tumor was not a prognostic factor for survival outcomes. We analyzed CXCR4 expression in CTCs by treatment arm, and Kaplan-Meier curves showed separation in PFS between treatment arms through the 4-month course of therapy, which did not reach statistical significance, for ≥6 CTCs or ≥7% CXCR4^+^ CTCs (Fig. 2a, b). This separation was not observed at 6 months. Since no differences in clinical endpoints were observed between treatment arms [23], data from both arms were combined for a larger sample size to evaluate biomarker prognostic value. This is a potential limitation of the exploratory post-hoc analyses; a prospective study is required to confirm CTC counts as prognostic of outcomes. It is notable that the combined sample size of our study (*N* = 89) is greater than those reported in several prior studies: 38 patients in Hiltermann et al. [10], and 24 patients in Naito et al. [12].

Our finding, that CXCR4 overexpression in tumors is not prognostic of outcomes in SCLC, contrasts with several recent meta-analyses of biomarker levels and lung cancer clinical outcomes. Liang et al. [32] showed high-level CXCR4 tumor expression (2037 patients) is related to poor prognosis in lung cancer patients, and Zhao et al. [33] showed that CXCR4 overexpression (11,032 patients) predicts unfavorable OS in lung cancer. Our analyses represent the first report we are aware of measuring CXCR4 expression in both tumor and CTCs, using the CELLSEARCH® platform. One previous report from Reckamp et al. [34] showed that low co-expression of CXCR4 and pan-cytokeratin in CTCs was prognostic of improved OS in NSCLC, based on flow cytometry analysis of peripheral blood from 16 patients; CXCR4 expression in NSCLC tumors was documented, but not further analyzed in addition to CTCs. Our observation, that elevated baseline CXCR4 expression in CTCs (≥7% CXCR4^+^ CTCs) is prognostic of worse PFS in ED-SCLC, whereas CXCR4 overexpression in tumor is not, has several possible explanations beyond study design. CTCs may better represent the cells shed by primary and metastatic tumors versus tumor tissue biopsied from a single site. CTC samples drawn immediately prior to start of the study may better represent disease status versus tumor biopsy that may have been obtained less proximal to start of the study. Additional prospective study is required for confirmation and clarity on CXCR4^+^ CTCs as a prognostic biomarker. Lastly, several isoforms of CXCR4 have been identified in cancer cell lines, including alternate splice variants [35, 36]. Our study did not differentiate between isoforms of CXCR4 detected in biopsied tumor and blood CTCs, the clinical relevance of which is not known.

In summary, positive CXCR4 expression in tumor tissue was not significantly prognostic of survival in 89 patients with ED-SCLC in the present analysis. Neither baseline CTC counts or CXCR4 expression in tumor tissue or CTCs were predictive of treatment response for CXCR4 peptide antagonist LY2510924 plus CE versus CE alone. However, in general, CTC enumeration and CXCR4 expression in CTCs are promising prognostic biomarkers for ED-SCLC at baseline and post-treatment, as evidenced in the literature. The feasibility of conducting biomarker, prognostic, and predictive analyses in future SCLC studies is facilitated by peripheral blood CTC collection as a liquid biopsy, which has the potential to replace technically difficult solid biopsies in this patient population.

## Electronic supplementary material

ESM 1 (DOCX 3313 kb)
