# Supplementary material for: Prognostic and predictive value of circulating tumor cells and CXCR4 expression as biomarkers for a CXCR4 peptide antagonist in combination with carboplatin-etoposide in small cell lung cancer: exploratory analysis of a phase II study
Source: Invest New Drugs. 2017 Mar 15;35(3):334–44. doi: 10.1007/s10637-017-0446-z (PMC5418321; doi:10.1007/s10637-017-0446-z)
Supplement: Supplementary file 1 — (DOCX 3313 kb) [file 10637_2017_446_MOESM1_ESM.docx]

**Prognostic and Predictive Value of Circulating Tumor Cells and CXCR4 Expression as Biomarkers for a CXCR4 Peptide Antagonist in Combination with Carboplatin-Etoposide in Small Cell Lung Cancer: Exploratory Analysis of a Phase II Study**

Ravi Salgia,^1^* R. Waide Weaver,^2^ Michael McCleod,^3^ John R. Stille,^4^ S. Betty Yan,^5^ Stephanie Roberson,^4^ John Polzer,^4^ Amy Flynt,^6^ Eyas Raddad,^4^ Victoria L. Peek,^5^ Sameera R. Wijayawardana,^5^ Suzane L. Um,^5^ Steve Gross,^7^ Mark C. Connelly,^7^ Carrie Morano,^7^ Madeline Repollet,^7^ Renouard Sanders,^7^ Kurt Baeten,^8^ David D’Haese,^8^ David R. Spigel^9^

^1^ City of Hope Comprehensive Cancer Center, Duarte, CA, USA

^2^ Florida Cancer Specialists, St. Petersburg, FL, USA

^3^ Florida Cancer Specialists, Fort Myers, FL, USA

^4^ The Chorus Group, Eli Lilly and Company, Indianapolis, IN, USA

^5^ Lilly Research Laboratories, Eli Lilly and Company, Indianapolis, IN, USA

^6^ PharPoint Research Inc., Durham, NC, USA

^7^ Janssen Diagnostics, Johnson and Johnson Company, Raritan, NJ, USA

^8^ Janssen Diagnostics, Janssen Pharmaceutica, Beerse, Belgium

^9^ Sarah Cannon Research Institute, Nashville, TN, USA

* **Corresponding author**:
Ravi Salgia, MD, PhD
City of Hope Comprehensive Cancer Center
1500 E. Duarte Road
Duarte, CA 91010-3000
Phone: (626) 471-9200
Fax: (626) 471-7322
Email: rsalgia@coh.org

# Supplementary Section

**Supplementary Materials and** **Methods**

**CTC assay for detection of CXCR4 expression**

Blood was collected into a CellSave preservative tube (Janssen Diagnostics, Raritan, NJ) at baseline (day 1 of cycle 1); cycle 1, day 7; cycle 2, day 1; and at 30-day follow-up after the last dose of study drug. Circulating tumor cells (CTCs) were isolated and enumerated using the CXC CELLSEARCH^®^ kit and CELLTRACKS^®^ AUTOPREP^®^ system according to the manufacturer’s instructions (Janssen Diagnostics LLC, Raritan, NJ) as described previously [1]. Samples were scanned and reviewed using the CELLTRACKS ANALYZER II^®^ and a modified version of the CELLTRACKS software (BioMarQ). Three of the 4 fluorescent BioMarQ channels were used to distinguish CTC from white blood cells: nuclear dye 4',6-diamidino-2-phenylindole (DAPI), anti-cytokeratin, and anti-CD45. The fourth channel was used to measure CXCR4 expression using an anti-CXCR4 phycoerythrin (PE)-labeled antibody. Positive and negative control cell lines for CXCR4 expression were included in each batch of clinical samples.

After CTC enumeration of each sample, CXCR4 expression was visually phenotyped as positive (CXCR4^+^) or negative (CXCR4^-^) by an operator (see the following assay details). Two patient samples in this study that were scored by an operator for CTC count and CXCR4 expression had >10,000 images for review; these samples were partially reviewed and scored. For 1 of these samples, CTC enumeration stopped at 5000 random images and the total number of CTCs was extrapolated based on the proportion of the partial CTC count to the total number of images available for review. CXCR4 expression was reported as the percent positivity of CTCs present in the 5000 images analyzed. The other large sample was reviewed up to 10,000 images; the same steps were followed for CTC extrapolation and CXCR4 expression.

For initial assay development, the Mouse/Rat CellCapture CTC kit (Janssen Diagnostics, Raritan, NJ) was used to screen for appropriate anti-CXCR4 antibodies, select positive and negative control tumor cells, and evaluate parameters for the fourth channel for CXCR4 detection. MDA‑MB‑231 cells transfected with CXCR4 were used for assay development for consistent CXCR4 staining. Cells were collected and resuspended in phosphate buffer saline (PBS) and counted with a Vi-CELL Cell Viability Analyzer (Beckman Coulter, Brea, CA). Approximately 500 cells were spiked into 0.06–0.1 mL of blood from athymic nude mice. Samples were processed according to the manufacturer’s instructions. Anti-human CXCR4 antibody (MAB171/clone 44708, R&D Systems, Minneapolis, MN) labeled with PE using the R-PE Labeling Kit‑SH (Dojindo Molecular Technologies, Inc., Rockville, MD) was added to the sample at the same time as nucleic acid dye, anti-CK fluorescein isothiocyanate (FITC) reagent, and permeabilization reagent. Samples were analyzed with a CELLTRACKS ANALYZER II (Janssen Diagnostics, Raritan, NJ) for CTC count and number of CTCs expressing CXCR4.

*CXCR4 phycoerythrin conjugation*

Further optimization and validation of this assay was conducted at the research and clinical laboratories at Janssen Diagnostics (Huntingdon Valley, PA). Lyophilized anti-CXCR4 antibody (clone 44708) was purchased in bulk from R&D Systems (Minneapolis, MN). The conjugation to PE was performed using 2 different methods.

The first method utilized the commercial R-PE Labeling Kit-SH (Dojindo Molecular Technologies, Inc., Rockville, MD) and the anti-CXCR4 antibody was labeled with PE using the manufacturer’s instructions. Briefly, the antibody was treated with a reducing agent to produce free sulfhydryl groups; then a reaction buffer containing maleimide groups was added to the reduced antibody, linking the PE to the reduced sulfhydryl groups.

The second method used Janssen’s proprietary hetero-bifunctional chemistry to link PE (ProZyme, Hayword, CA) to the CXCR4 antibody. In this process, PE was activated using sulfo-SMCC (Pierce, Rockford, IL) and antibodies were reduced using dithiothreitol (DTT) (Sigma, St. Louis, MO) and then separated from free DTT by gel filtration chromatography. The activated PE was covalently coupled to the reduced antibodies, and the reaction was quenched with N-ethylmaleimide (Fluka, Buchs, Germany) for 20 minutes. The PE-conjugated antibodies were purified with a size-exclusion Tosoh TSKgel G3000SW column on an AKTA explorer FPLC system (GE Healthcare Bio-Sciences, Piscataway, NJ).

*CXCR4 phycoerythrin evaluation on cell lines by flow cytometry*

Cell lines with either null CXCR4 expression (MDA-MB-231) or low/medium CXCR4 expression (COLO-205 and DU4475) were purchased from ATCC (Manassas, VA). A stably-transfected cell line, MDA-MB‑231-CXCR4, containing a vector encoding human CXCR4 and expressing high levels of CXCR4 was provided by Eli Lilly and Company (Indianapolis, IN). The different anti-CXCR4 antibody PE conjugates were initially titrated on 4 cell lines and then analyzed with a FACSCalibur (BD Biosciences, San Jose, CA) flow cytometer. The adherent cell lines, MDA-MB-231, and MDA-MB-231-CXCR4 were trypsinized and harvested from cell culture. The cells were washed once and resuspended with supplemented RPMI medium. The suspension cell lines, DU4475 and COLO-205, were collected, washed, and resuspended in supplemented RPMI medium. Cell concentration and viability were determined using Trypan blue exclusion and hemocytometer counting; cell viability was consistently >90%. The remaining MDA-MB‑231-CXCR4 cells were then fixed in either 0.45% paraformaldehyde or neutral buffered formalin. Fixation was necessary as these cells were mycoplasma positive and could not be continually grown in the culture facility. The fixed cells were tested for CXCR4 expression.

Cells were typically stained with the direct antibody conjugates for 20 minutes in a final volume of 200 µL at a cell concentration not exceeding 10^6^ cells/mL. Cells were washed once with 3 mL of PBS and resuspended to a final volume of 300 µL in CellFix (Janssen Diagnostics, Huntingdon Valley, PA). Approximately 2000–3000 cells were acquired on the FACSCalibur flow cytometer for analysis. Cells were then stored overnight in CellSave (Janssen Diagnostics, Huntingdon Valley, PA) to monitor the effect of CellSave on CXCR4 expression. CellSave and the CellSave preservative tube contain an optimized preservative that stabilizes cells, particularly CTCs, for up to 96 hours at room temperature. This allows shipment of samples to remote locations for analysis and improves the reproducibility and reliability of CTC analysis.

*Anti-CXCR4 antibody-phycoerythrin evaluation on cell lines by CELLSEARCH*

The CELLSEARCH platform consists of an automated sample preparation instrument known as the CELLTRACKS AUTOPREP and the CELLTRACKS ANALYZER II. The CELLTRACKS ANALYZER II is a semi-automated scanning fluorescence microscope system used to count and characterize rare cells that have been immunomagnetically selected and placed within a cartridge. A grayscale charge-coupled device camera was used to image each of 4 color fluorescence channels. For testing on CELLSEARCH, cells were harvested as described previously and spiked into 7.5 mL blood collected in CellSave preservative tubes, stored at room temperature overnight, and processed the following day. Spiked samples were run using the RUO CELLSEARCH CXC kit (Janssen Diagnostics, Raritan, NJ). This kit consists of ferrofluids coated with epithelial cell-specific EpCAM antibodies to immunomagnetically enrich epithelial cells; a mixture of 2 fluorescein-conjugated antibodies that bind to cytokeratins 8, 18, and 19; an antibody to CD45 conjugated to allophycocyanin (APC); DAPI; and buffers to wash, permeabilize, and resuspend cells.

To determine the optimum camera exposure or integration time for the PE filter used for CXCR4 expression on the CELLTRACKS ANALYZER II, the camera integration time was tested over the range of 0.05–0.2 second. This range was used for determining background in the PE channel using different cell lines first without the addition of the anti-CXCR4 antibody-PE marker, and again in the presence of the marker. For the final validated clinical assay, 0.05 second was selected as the optimum integration time. The percentage of cells staining positive for CXCR4 was calculated; once optimized, the assay was transferred to Janssen Diagnostic’s clinical laboratory. Representative images of tumor cell lines with null (MDA-MB-231) or positive (DU4475) CXCR4 expression in this assay are shown in Supplementary Figure S1a–d. Representative images of a tumor cell line stably transfected with CXCR4 (MDA-MB‑231) in this assay, with or without anti-CXCR4 PE-labeled antibody labeling, are shown in Supplementary Figure S1e–f. A representative image of CXCR4^+^ CTCs from a SCLC patient in this assay is shown in Supplementary Figure S1g.

*Patient sample testing*

One 10-mL sample tube was received for most collection time points. A total of 7.5 mL blood was used to enumerate CTCs and CXCR4 expression. Blood was collected in CellSave preservative tubes and maintained at room temperature. For any tube with <7.5 mL blood, if a second tube for the patient was obtained with <7.5 mL blood then tubes were pooled to achieve adequate testing volume. Blood samples were processed on the CELLSEARCH AUTOPREP with a CELLSEARCH CXC kit and anti-CXCR4 antibody-PE marker (Janssen Diagnostics, Huntingdon Valley, PA) for CXCR4^+^ CTC expression. The CXCR4 reagent was placed in the reagent carrousel and added by the instrument as a marker reagent. CELLSEARCH CXC Control kit and fixed MDA-231-CXCR4 cells were processed as quality controls.

**Table S1** Correlation of baseline tumor CXCR4 expression versus CXCR4 expression in CTCs (%CXCR4^+^ CTC)

|  | **LY2510924 + CE**  **(*N*=47)** | **CE**  **(*N*=42)** | **Total**  **(*N*=89)** |
| --- | --- | --- | --- |
| Patients with values, *n* | 29 | 26 | 55 |
| Pearson correlation (r), 95% CI | 0.461 (0.106, 0.704) | 0.373 (-0.024, 0.660) | 0.423 (0.174, 0.616) |
| P-value* | 0.011 | 0.06 | 0.001 |

*CE* carboplatin-etoposide, *CI* confidence interval, *CTCs* circulating tumor cells, *CXCR4* chemokine (C-X-C motif) receptor 4, *N* number of patients

* P-value from Pearson correlation test

**Table S2** Determination of biomarker optimum cutoffs using 6-month PFS and 11.5-month OS for the overall study population

| **Biomarker** | **Number of Patients** | **AUC (95% CI)** | **P-Value^a^** | **Optimum Cutoff** | **Sensitivity^b^ (%)** | **Specificity^c^ (%)** | **Positive Predictive Value^d^ (%)** | **Negative Predictive Value^e^  (%)** |
| --- | --- | --- | --- | --- | --- | --- | --- | --- |
|  |  |  |  | **PFS** | |  |  |  |
| Baseline |  |  |  |  |  |  |  |  |
| CXCR4^+^ tumor tissue | 69 | 0.631 (0.496, 0.765) | 0.122 | 210 | 54.8 | 70.4 | 74.2 | 50.0 |
| CTC count | 78 | 0.607 (0.471, 0.742) | 0.284 | 6 | 84.3 | 37.0 | 71.7 | 55.6 |
| %CXCR4^+^ CTCs | 70 | 0.702 (0.577, 0.828) | 0.011 | 7 | 75.6 | 60.0 | 77.3 | 57.7 |
| Cycle 1, day 7 |  |  |  |  |  |  |  |  |
| CTC count | 62 | 0.530 (0.370, 0.690) | 0.891 | 6 | 42.9 | 60.0 | 69.2 | 33.3 |
| %CXCR4^+^ CTCs | 56 | 0.488 (0.380, 0.596) | 0.794 | 7 | 89.2 | 10.5 | 66.0 | 33.3 |
| Cycle 2, day 1 |  |  |  |  |  |  |  |  |
| CTC count | 61 | 0.634 (0.501, 0.766) | 0.256 | 6 | 45.7 | 76.9 | 72.7 | 51.3 |
| %CXCR4^+^ CTCs | 57 | 0.558 (0.434, 0.681) | 0.611 | 7 | 29.4 | 78.3 | 66.7 | 42.9 |
|  |  |  | **OS** | | |  |  |  |
| Baseline |  |  |  |  |  |  |  |  |
| CXCR4^+^ tumor tissue | 69 | 0.513 (0.372, 0.654) | 0.737 | 210 | 57.8 | 45.8 | 66.7 | 36.7 |
| CTC count | 78 | 0.549 (0.408, 0.689) | 0.737 | 6 | 82.0 | 32.1 | 68.3 | 50.0 |
| %CXCR4^+^ CTCs | 70 | 0.563 (0.425, 0.702) | 0.391 | 7 | 65.2 | 41.7 | 68.2 | 38.5 |
| Cycle 1, day 7 |  |  |  |  |  |  |  |  |
| CTC count | 62 | 0.425 (0.269, 0.581) | 0.307 | 6 | 55.0 | 31.8 | 59.5 | 28.0 |
| %CXCR4^+^ CTCs | 56 | 0.518 (0.408, 0.628) | 0.564 | 7 | 91.7 | 15.0 | 66.0 | 50.0 |
| Cycle 2, day 1 |  |  |  |  |  |  |  |  |
| CTC count | 61 | 0.674 (0.549, 0.799) | 0.296 | 6 | 47.2 | 80.0 | 77.3 | 51.3 |
| %CXCR4^+^ CTCs | 57 | 0.569 (0.444, 0.694) | 0.651 | 7 | 30.6 | 81.0 | 73.3 | 40.5 |

*AUC* area under the curve, *CI* confidence interval, *CTCs* circulating tumor cells, *CXCR4* chemokine (C-X-C motif) receptor 4, *n* number of patients in a category, *OS* overall survival, *PFS* progression-free survival
^a^ P-value from chi-square test of null hypothesis that regression coefficient was 0; the 95% CI for AUC was the Wald confidence limits; this was an asymptotic CI
^b^ Sensitivity: % of patients with value ≥ cutoff who were either deceased or had disease progression at 6 months out of patients who were either deceased or had disease progression at 6 months

^c^ Specificity: % of patients with value < cutoff alive without disease progression at 6 months out of patients alive without disease progression at 6 months

^d^ Positive predictive value: % of patients with value ≥ cutoff who were either deceased or had disease progression at 6 months out of patients with value ≥ cutoff

^e^ Negative predictive value: % of patients with value < cutoff alive without disease progression at 6 months out of patients with value < cutoff

**Table S3** Summary of biomarker cutoff levels by visit* and treatment arm

|  | **LY2510924 + CE (*N*=47)** | **CE (*N*=42)** | **Total (*N*=89)** |
| --- | --- | --- | --- |
| **CXCR4^+^ in Tumor Tissue** | | | |
| Baseline |  |  |  |
| <210 CXCR4^+^ in tumor tissue, *n/N* (%) | 20/36 (55.6) | 18/33 (54.5) | 38/69 (55.1) |
| ≥210 CXCR4^+^ in tumor tissue, *n/N* (%) | 16/36 (44.4) | 15/33 (45.5) | 31/69 (44.9) |
| ≥210 CXCR4^+^ in tumor tissue, 95% CI | 27.9, 61.9 | 28.1, 63.6 | 32.9, 57.4 |
| **CTC Count** | | | |
| Baseline |  |  |  |
| <6 CTC count, *n/N* (%) | 8/25 (32.0) | 7/21 (33.3) | 15/46 (32.6) |
| ≥6 CTC count, *n/N* (%) | 17/25 (68.0) | 14/21 (66.7) | 31/46 (67.4) |
| ≥6 CTC count, 95% CI | 46.5, 85.1 | 43.0, 85.4 | 52.0, 80.5 |
| Cycle 1, day 7 |  |  |  |
| <6 CTC count, *n/N* (%) | 13/25 (52.0) | 13/21 (61.9) | 26/46 (56.5) |
| ≥6 CTC count, *n/N* (%) | 12/25 (48.0) | 8/21 (38.1) | 20/46 (43.5) |
| ≥6 CTC count, 95% CI | 27.8, 68.7 | 18.1, 61.6 | 28.9, 58.9 |
| Cycle 2, day 1 |  |  |  |
| <6 CTC count, *n/N* (%) | 16/25 (64.0) | 14/21 (66.7) | 30/46 (65.2) |
| ≥6 CTC count, *n/N* (%) | 9/25 (36.0) | 7/21 (33.3) | 16/46 (34.8) |
| ≥6 CTC count, 95% CI | 18.0, 57.5 | 14.6, 57.0 | 21.4, 50.2 |
| **%CXCR4^+^ CTCs** | | | |
| Baseline |  |  |  |
| <7% CXCR4^+^ CTCs, *n/N* (%) | 12/23 (52.2) | 6/19 (31.6) | 18/42 (42.9) |
| ≥7% CXCR4^+^ CTCs, *n/N* (%) | 11/23 (47.8) | 13/19 (68.4) | 24/42 (57.1) |
| ≥7% CXCR4^+^ CTCs, 95% CI | 26.8, 69.4 | 43.4, 87.4 | 41.0, 72.3 |
| Cycle 1, day 7 |  |  |  |
| <7% CXCR4^+^ CTCs, *n/N* (%) | 23/23 (100.0) | 12/19 (63.2) | 35/42 (83.3) |
| ≥7% CXCR4^+^ CTCs, *n/N* (%) | 0/23 | 7/19 (36.8) | 7/42 (16.7) |
| ≥7% CXCR4^+^ CTCs, 95% CI | 0.0, 14.8 | 16.3, 61.6 | 7.0, 31.4 |
| Cycle 2, day 1 |  |  |  |
| <7% CXCR4^+^ CTCs, *n/N* (%) | 16/23 (69.6) | 16/19 (84.2) | 32/42 (76.2) |
| ≥7% CXCR4^+^ CTCs, *n/N* (%) | 7/23 (30.4) | 3/19 (15.8) | 10/42 (23.8) |
| ≥7% CXCR4^+^ CTCs, 95% CI | 13.2, 52.9 | 3.4, 39.6 | 12.1, 39.5 |

*CE* carboplatin-etoposide, *CI* confidence interval for higher-risk group, *CTCs* circulating tumor cells, *CXCR4* chemokine (C-X-C motif) receptor 4, *IHC*, *N* number of patients, *n* number of patients in a category

* Denominators include only those patients within the treatment group who had available biomarker data at all visits

**Table S4** Predictive value of biomarkers for progression-free survival and overall survival (4 months and 6 months) by treatment arm

|  | **Baseline CXCR4^+^ in Tumor Tissue (H-score)** | | | | | | | | | |
| --- | --- | --- | --- | --- | --- | --- | --- | --- | --- | --- |
|  | **LY2510924 + CE** | | | |  | **CE** | | | | |
|  | **<210**  **(*N*=20)** | **≥210**  **(*N*=16)** | **Total**  **(*N*=36)** | |  | **<210**  **(*N*=18)** | **≥210**  **(*N*=15)** | | **Total**  **(*N*=33)** | |
| **PFS** |  |  |  | |  |  |  | |  | |
| Kaplan-Meier estimate (mos) |  |  |  | |  |  |  | |  | |
| Mean (SD) | 6.7 (0.6) | 5.1 (0.4) | 6.1 (0.5) | |  | 6.5 (0.8) | 5.8 (0.9) | | 6.2 (0.6) | |
| Median (95% CI) | 6.1 (4.8, 7.3) | 5.5 (3.6, 6.3) | 6.1 (4.8, 6.3) | |  | 6.4 (3.1, 7.9) | 5.8 (2.8, 7.0) | | 5.9 (4.2, 6.7) | |
| HR <210 vs ≥210 (95% CI) 4 mos |  |  | 0.34 (0.06, 1.83) | |  | \|  \| \| --- \| |  | | 0.66 (0.18, 2.46) | |
| P-value through 4 mos* |  |  | 0.184 | |  |  |  | | 0.531 | |
| HR <210 vs ≥210 (95% CI) 6 mos |  |  | 0.53 (0.18, 1.51) | |  |  |  | | 0.65 (0.24, 1.80) | |
| P-value through 6 mos* |  |  | 0.226 | |  |  |  | | 0.401 | |
| **OS** |  |  |  | |  |  |  | |  | |
| Kaplan-Meier estimate (mos) |  |  |  | |  |  |  | |  | |
| Mean (SD) | 9.7 (0.9) | 8.4 (0.9) | 9.1 (0.6) | |  | 11.3 (1.4) | 11.8 (2.0) | | 11.6 (1.2) | |
| Median (95% CI) | 9.9 (6.1, 13.7) | 7.4 (4.3, 10.7) | 9.2 (6.4, 12.3) | |  | 11.1 (7.6, 13.4) | 12.5 (3.6, 20.1) | | 11.2 (7.6, 13.4) | |
| HR <210 vs ≥210 (95% CI) 4 mos |  |  | 0.38 (0.03, 4.18) | |  |  |  | | 0.40 (0.07, 2.16) | |
| P-value through 4 mos* |  |  | 0.410 | |  |  |  | | 0.268 | |
| HR <210 vs ≥210 (95% CI) 6 mos |  |  | 0.89 (0.20, 3.97) | |  |  |  | | 0.46 (0.11, 1.94) | |
| P-value through 6 mos* |  |  | 0.876 | |  |  |  | | 0.280 | |
|  | **CTC Count (Baseline)** | | | | | | | | | |
|  | **LY2510924 + CE** | | | |  | **CE** | | | | |
|  | **CTC <6**  **(*N*=11)** | **CTC ≥6**  **(*N*=31)** | **Total**  **(*N*=42)** | |  | **CTC <6**  **(*N*=7)** | **CTC ≥6**  **(*N*=29)** | | **Total**  **(*N*=36)** | |
| **PFS** |  |  |  | |  |  |  | |  | |
| Kaplan-Meier estimate (mos) |  |  |  | |  |  |  | |  | |
| Mean (SD) | 6.7 (0.9) | 5.9 (0.51) | 6.2 (0.5) | |  | 8.0 (1.3) | 5.1 (0.5) | | 5.7 (0.6) | |
| Median (95% CI) | 7.3 (2.8, NA) | 5.6 (4.4, 6.1) | 6.1 (4.8, 6.3) | |  | 6.60 (4.2, 12.7) | 4.83 (3.06, 6.3) | | 5.78 (3.9, 6.5) | |
| HR <6 vs ≥6 (95% CI) 4 mos |  |  | 1.03 (0.20, 5.11) | |  |  |  | | 0.00 (0.00, NE) | |
| P-value through 4 mos* |  |  | 0.972 | |  |  |  | | 0.049 | |
| HR <6 vs ≥6 (95% CI) 6 mos |  |  | 0.42 (0.10, 1.85) | |  |  |  | | 0.30 (0.07, 1.32) | |
| P-value through 6 mos* |  |  | 0.236 | |  |  |  | | 0.092 | |
| **OS** |  |  |  | |  |  |  | |  | |
| Kaplan-Meier estimate (mos) |  |  |  | |  |  |  | |  | |
| Mean (SD) | 10.9 (1.2) | 8.7 (0.6) | 9.3 (0.6) | |  | 16.5 (2.7) | 10.8 (1.3) | | 11.9 (1.2) | |
| Median (95% CI) | 13.1 (4.3, NA) | 9.2 (6.4, 10.8) | 10.0 (6.6, 12.3) | |  | 20.1 (5.0, 20.1) | 9.3 (6.0, 13.4) | | 11.6 (7.6, 19.1) | |
| HR <6 vs ≥6 (95% CI) 4 mos |  |  | 0.93 (0.10, 8.95) | |  |  |  | | 0.00 (0.00, NE) | |
| P-value through 4 mos* |  |  | 0.950 | |  |  |  | | 0.169 | |
| HR <6 vs ≥6 (95% CI) 6 mos |  |  | 1.01 (0.20, 5.01) | |  |  |  | | 0.40 (0.05, 3.17) | |
| P-value through 6 mos* |  |  | 0.990 | |  |  |  | | 0.37 | |
|  | **CTC Count (Cycle 2, Day 1)** | | | | | | | | | |
|  | **LY2510924 + CE** | | | |  | **CE** | | | | |
|  | **CTC <6**  **(*N*=22)** | **CTC ≥6**  **(*N*=12)** | **Total**  **(*N*=34)** | |  | **CTC <6**  **(*N*=17)** | **CTC ≥6**  **(*N*=10)** | | **Total**  **(*N*=27)** | |
| **PFS** |  |  |  | |  |  |  | |  | |
| Kaplan-Meier estimate (mos) |  |  |  | |  |  |  | |  | |
| Mean (SD) | 7.1 (0.708) | 5.4 (0.842) | 6.5 (0.575) | |  | 7.7 (0.794) | 4.6 (0.481) | | 6.6 (0.610) | |
| Median (95% CI) | 6.6 (4.8, 10.6) | 5.5 (2.7, 6.2) | 6.1 (4.8, 7.3) | |  | 7.0 (5.6, 9.5) | 4.6 (2.6, 6.4) | | 6.0 (4.6, 7.0) | |
| HR <6 vs ≥6 (95% CI) 4 mos |  |  | 0.26 (0.05, 1.41) | |  |  |  | | 0.26 (0.05, 1.43) | |
| P-value through 4 mos* |  |  | 0.093 | |  |  |  | | 0.095 | |
| HR <6 vs ≥6 (95% CI) 6 mos |  |  | 0.38 (0.12, 1.21) | |  |  |  | | 0.27 (0.08, 0.86) | |
| P-value through 6 mos* |  |  | 0.089 | |  |  |  | | 0.018 | |
| **OS** |  |  |  | |  |  |  | |  | |
| Kaplan-Meier estimate (mos) |  |  |  | |  |  |  | |  | |
| Mean (SD) | 10.6 (0.6) | 7.7 (1.1) | 9.6 (0.6) | |  | 15.7 (1.5) | 8.8 (1.3) | | 13.5 (1.3) | |
| Median (95% CI) | 11.7 (9.2, 13.1) | 6.4 (4.3, 12.3) | 10.4 (6.9, 12.3) | |  | 19.5 (9.8, NA) | 8.8 (2.9, 13.4) | | 12.7 (9.2, 20.0) | |
| HR <6 vs ≥6 (95% CI) 4 mos |  |  | 0.56 (0.04, 8.93) | |  |  |  | | 0.26 (0.02, 2.91) | |
| P-value through 4 mos* |  |  | 0.676 | |  |  |  | | 0.242 | |
| HR <6 vs ≥6 (95% CI) 6 mos |  |  | 0.13 (0.02, 1.17) | |  |  |  | | 0.35 (0.06, 2.09) | |
| P-value through 6 mos* |  |  | 0.031 | |  |  |  | | 0.227 | |
|  | **%CXCR4^+^ CTCs (Baseline)** | | | | | | | | | |
|  | **LY2510924 + CE** | | | |  | **CE** | | | | |
|  | **%CXCR4^+^ CTC <7 (*N*=17)** | **%CXCR4^+^ CTC ≥7 (*N*=20)** | | **Total**  **(*N*=37)** |  | **%CXCR4^+^ CTC <7 (*N*=9)** | | **%CXCR4^+^ CTC ≥7 (*N*=24)** | | **Total**  **(*N*=33)** |
| **PFS** |  |  | |  |  |  | |  | |  |
| Kaplan-Meier estimate (mos) |  |  | |  |  |  | |  | |  |
| Mean (SD) | 6.7 (0.8) | 5.5 (0.4) | | 6.2 (0.5) |  | 7.2 (1.2) | | 4.9 (0.5) | | 5.7 (0.6) |
| Median (95% CI) | 6.2 (3.1, 8.7) | 5.5 (3.9, 6.1) | | 6.1 (4.9, 6.3) |  | 6.6 (3.0 12.7) | | 4.8 (3.1, 6.2) | | 5.8 (3.8, 6.3) |
| HR <7 vs ≥7 (95% CI) 4 mos |  |  | | 1.18 (0.30, 4.74) |  |  | |  | | 0.49 (0.11, 2.25) |
| P-value through 4 mos* |  |  | | 0.811 |  |  | |  | | 0.341 |
| HR <7 vs ≥7 (95% CI) 6 mos |  |  | | 0.55 (0.18, 1.64) |  |  | |  | | 0.40 (0.11, 1.39) |
| P-value through 6 mos* |  |  | | 0.274 |  |  | |  | | 0.134 |
| **OS** |  |  | |  |  |  | |  | |  |
| Kaplan-Meier estimate (mos) |  |  | |  |  |  | |  | |  |
| Mean (SD) | 9.3 (1.0) | 8.7 (0.8) | | 9.0 (0.7) |  | 9.3 (1.3) | | 11.4 (1.4) | | 12.0 (1.3) |
| Median (95% CI) | 10.4 (4.7, NE) | 8.8 (6.2, 11.7) | | 9.2 (6.4, 12.3) |  | NE (3.1, NE) | | 9.3 (6.1, 19.1) | | 9.8 (7.6, 20.1) |
| HR <7 vs ≥7 (95% CI) 4 mos |  |  | | 1.24 (0.17, 8.77) |  |  | |  | | 0.52 (0.06, 4.43) |
| P-value through 4 mos* |  |  | | 0.831 |  |  | |  | | 0.540 |
| HR <7 vs ≥7 (95% CI) 6 mos |  |  | | 1.21 (0.30, 4.83) |  |  | |  | | 1.29 (0.32, 5.15) |
| P-value through 6 mos* |  |  | | 0.789 |  |  | |  | | 0.720 |
|  | **%CXCR4^+^ CTCs (Cycle 2, Day 1)** | | | | | | | | | |
|  | **LY2510924 + CE** | | | |  | **CE** | | | | |
|  | **%CXCR4^+^ CTC <7 (*N*=24)** | **%CXCR4^+^ CTC ≥7 (*N*=9)** | | **Total**  **(*N*=33)** |  | **%CXCR4^+^ CTC <7**  **(*N*=18)** | | **%CXCR4^+^ CTC ≥7**  **(*N*=6)** | | **Total**  **(*N*=24)** |
| **PFS** |  |  | |  |  |  | |  | |  |
| Kaplan-Meier estimate (mos) |  |  | |  |  |  | |  | |  |
| Mean (SD) | 6.7 (0.7) | 5.0 (0.6) | | 6.4 (0.6) |  | 6.6 (0.7) | | 4.8 (0.7) | | 6.2 (0.6) |
| Median (95% CI) | 6.2 (4.8, 8.3) | 5.6 (1.4, 6.2) | | 6.1 (4.8, 7.3) |  | 6.0 (4.2, 7.9) | | 4.9 (2.9, 6.5) | | 5.9 (4.2, 6.5) |
| HR <7 vs ≥7 (95% CI) 4 mos |  |  | | 0.78 (0.14, 4.25) |  |  | |  | | 0.32 (0.06, 1.58) |
| P-value through 4 mos* |  |  | | 0.770 |  |  | |  | | 0.140 |
| HR <7 vs ≥7 (95% CI) 6 mos |  |  | | 0.77 (0.23, 2.55) |  |  | |  | | 0.56 (0.17, 1.87) |
| P-value through 6 mos* |  |  | | 0.665 |  |  | |  | | 0.338 |
| **OS** |  |  | |  |  |  | |  | |  |
| Kaplan-Meier estimate (mos) |  |  | |  |  |  | |  | |  |
| Mean (SD) | 10.0 (0.7) | 7.9 (1.4) | | 9.5 (0.7) |  | 13.6 (1.6) | | 9.2 (1.7) | | 13.0 (1.4) |
| Median (95% CI) | 10.8 (7.4, 12.3) | 6.4 (3.6, 13.7) | | 10.0 (6.7, 12.3) |  | 12.7 (9.2, NE) | | 10.5 (2.9, NE) | | 12.5 (8.4, 20.1) |
| HR <7 vs ≥7 (95% CI) 4 mos |  |  | | 0.38 (0.02, 6.13) |  |  | |  | | 0.60 (0.06, 6.65) |
| P-value through 4 mos* |  |  | | 0.481 |  |  | |  | | 0.676 |
| HR <7 vs ≥7 (95% CI) 6 mos |  |  | | 0.24 (0.04, 1.42) |  |  | |  | | 1.24 (0.14, 11.10) |
| P-value through 6 mos* |  |  | | 0.086 |  |  | |  | | 0.847 |

*CE* carboplatin-etoposide, *CI* confidence interval; *CTC* circulating tumor cell, *CXCR4* chemokine (C-X-C motif) receptor 4, *HR* hazard ratio, *mos* months, *N* number of patients, *n* number of patients in a category, *NE* not evaluable, *OS* overall survival, *PFS* progression-free survival, *SD* standard deviation
* P-value from a log-rank test

**Table S5** Prognostic value of biomarkers at baseline and post-baseline for PFS and OS for the overall study population

|  | **CXCR4^+^ Tumor Tissue H-score (Baseline)** | | |
| --- | --- | --- | --- |
|  | **<210  (*N*=38)** | **≥210  (*N*=31)** | **Total  (*N*=69)** |
| **PFS** |  |  |  |
| Kaplan-Meier estimate (mos) |  |  |  |
| Mean (SD) | 6.6 (0.5) | 5.5 (0.6) | 6.2 (0.4) |
| Median (95% CI) | 6.1 (5.6, 6.7) | 5.6 (3.9, 6.2) | 6.1 (5.5, 6.3) |
| HR <210 vs ≥210 (95% CI) |  |  | 0.65 (0.37, 1.13) |
| P-value* |  |  | 0.125 |
| **OS** |  |  |  |
| Kaplan-Meier estimate (mos) |  |  |  |
| Mean (SD) | 11.0 (0.9) | 10.3 (1.2) | 10.7 (0.7) |
| Median (95% CI) | 10.0 (8.3, 12.3) | 9.3 (6.0, 12.7) | 9.8 (7.6, 12.3) |
| HR <210 vs ≥210 (95% CI) |  |  | 0.93 (0.53, 1.65) |
| P-value* |  |  | 0.815 |
|  | **CTC Count (Baseline)** | | |
|  | **<6  (*N*=18)** | **≥6  (*N*=60)** | **Total  (*N*=78)** |
| **PFS** |  |  |  |
| Kaplan-Meier estimate (mos) |  |  |  |
| Mean (SD) | 7.7 (0.95) | 5.5 (0.37) | 6.0 (0.38) |
| Median (95% CI) | 7.3 (5.9, 12.7) | 5.6 (4.4, 6.1) | 5.9 (4.8, 6.2) |
| HR <6 vs ≥6 (95% CI) |  |  | 0.47 (0.24, 0.92) |
| P-value* |  |  | 0.024 |
| **OS** |  |  |  |
| Kaplan-Meier estimate (mos) |  |  |  |
| Mean (SD) | 14.7 (1.65) | 9.9 (0.75) | 11.0 (0.72) |
| Median (95% CI) | 20.0 (10.4, 20.1) | 9.2 (6.6, 10.8) | 10.0 (8.4, 12.3) |
| HR <6 vs ≥6 (95% CI) |  |  | 0.41 (0.19, 0.88) |
| P-value* |  |  | 0.017 |
|  | **CTC Count (Cycle 2, Day 1)** | | |
|  | **<6  (*N*=39)** | **≥6  (*N*=22)** | **Total  (*N*=61)** |
| **PFS** |  |  |  |
| Kaplan-Meier estimate (mos) |  |  |  |
| Mean (SD) | 7.6 (0.6) | 5.1 (0.5) | 6.6 (0.4) |
| Median (95% CI) | 6.6 (6.0, 8.7) | 4.8 (3.1, 6.1) | 6.1 (5.5, 6.6) |
| HR <6 vs ≥6 (95% CI) |  |  | 0.38 (0.21, 0.70) |
| P-value* |  |  | 0.001 |
| **OS** |  |  |  |
| Kaplan-Meier estimate (mos) |  |  |  |
| Mean (SD) | 13.9 (1.0) | 8.2 (0.9) | 12.0 (0.8) |
| Median (95% CI) | 12.7 (10.4, 19.5) | 6.7 (4.7, 12.3) | 11.7 (9.2, 12.7) |
| HR <6 vs ≥6 (95% CI) |  |  | 0.35 (0.18, 0.67) |
| P-value* |  |  | 0.001 |
|  | **%CXCR4^+^ CTCs (Baseline)** | | |
|  | **<7%  (*N*=26)** | **≥7%  (*N*=44)** | **Total  (*N*=70)** |
| **PFS** |  |  |  |
| Kaplan-Meier estimate (mos) |  |  |  |
| Mean (SD) | 6.9 (0.7) | 5.3 (0.4) | 6.0 (0.4) |
| Median (95% CI) | 6.3 (5.6, 8.3) | 5.5 (4.2, 6.1) | 5.9 (4.8, 6.2) |
| HR <7% vs ≥7% (95% CI) |  |  | 0.53 (0.29, 0.95) |
| P-value* |  |  | 0.029 |
| **OS** |  |  |  |
| Kaplan-Meier estimate (mos) |  |  |  |
| Mean (SD) | 9.5 (0.81) | 10.3 (0.94) | 11.0 (0.80) |
| Median (95% CI) | 11.7 (6.1, NA) | 9.2 (6.7, 12.3) | 9.3 (7.4, 12.3) |
| HR <7% vs ≥7% (95% CI) |  |  | 0.69 (0.37, 1.30) |
| P-value* |  |  | 0.247 |
|  | **%CXCR4^+^ CTCs (Cycle 2, Day 1)** | | |
|  | **<7%  (*N*=42)** | **≥7%  (*N*=15)** | **Total  (*N*=57)** |
| **PFS** |  |  |  |
| Kaplan-Meier estimate (mos) |  |  |  |
| Mean (SD) | 6.7 (0.50) | 5.0 (0.43) | 6.4 (0.42) |
| Median (95% CI) | 6.1 (4.9, 7.0) | 5.7 (3.1, 6.4) | 6.0 (4.9, 6.4) |
| HR <7% vs ≥7% (95% CI) |  |  | 0.69 (0.35, 1.35) |
| P-value* |  |  | 0.273 |
| **OS** |  |  |  |
| Kaplan-Meier estimate (mos) |  |  |  |
| Mean (SD) | 12.5 (1.0) | 8.6 (1.1) | 11.7 (0.8) |
| Median (95% CI) | 11.7 (9.3, 13.1) | 8.4 (4.3, 13.7) | 10.8 (8.8, 12.5) |
| HR <7% vs ≥7% (95% CI) |  |  | 0.57 (0.28, 1.16) |
| P-value* |  |  | 0.113 |

*CI* confidence interval, *CTCs* circulating tumor cells, *CXCR4* chemokine (C-X-C motif) receptor 4, *HR* hazard ratio, *mos* months, *N* total number of patients, *OS* overall survival, *PFS* progression-free survival, *SD* standard deviation
* P-value from a log-rank test

# Supplementary Figure Legends

**Supplementary Fig. S1** Representative images of CXCR4 expression in CTCs using an optimized detection method (no anti-CXCR4 antibody-PE added to assay: a, c, e; anti-CXCR4 antibody-PE added to assay: b, d, f) **a,b** MDA-MB-231 cells with low-level CXCR4 expression were spiked into and recovered from blood as CTCs (0% and 1% of recovered CTCs were positive for CXCR4 expression with and without anti-CXCR4 antibody-PE, respectively); **c,d** DU4475 cells with intermediate-level CXCR4 expression were spiked into and recovered from blood as CTCs (0% and 54% of recovered CTCs were positive for CXCR4 expression with and without anti-CXCR4 antibody-PE, respectively); **e,f** MDA-MB-231-CXCR4 cells (stably transfected with CXCR4) with intermediate- to high-level CXCR4 expression were spiked into and recovered from blood as CTCs (0% and 61% of recovered CTCs were positive for CXCR4 expression with and without anti-CXCR4 antibody-PE, respectively); **g** 2024 CTCs were recovered from a 7.5 mL-blood sample from a SCLC patient; 55% of CTCs were positive for CXCR4 expression (representative image of CXCR4^+^ CTCs is shown)

**Supplementary Fig. S2 a** Correlation plot of baseline tumor tissue CXCR4 expression (H-score) versus baseline %CXCR4^+^ CTCs; **b** Receiver operating characteristic (ROC) curve for %CXCR4^+^ CTCs at baseline as a predictor of PFS at 6 months; **c** ROC curve for change in %CXCR4^+^ CTCs from baseline to cycle 1, day 7 as a predictor of PFS at 6 months; **d** ROC curve for tumor tissue CXCR4 expression (H-score) at baseline as a predictor of PFS at 6 months;  **e** ROC curve for CTC counts at baseline as a predictor of PFS at 6 months; **f** ROC curve for CTC counts at cycle 1, day 7 as a predictor of PFS at 6 months

*CE* carboplatin-etoposide, *CTC* circulating tumor cell, *CXCR4* chemokine (C-X-C motif) receptor 4, *LY* LY2510924

**e**

**b**

**d**

**f**

**c**

**a**

**Fig. S1**

**g**

**Fig. S2
a**


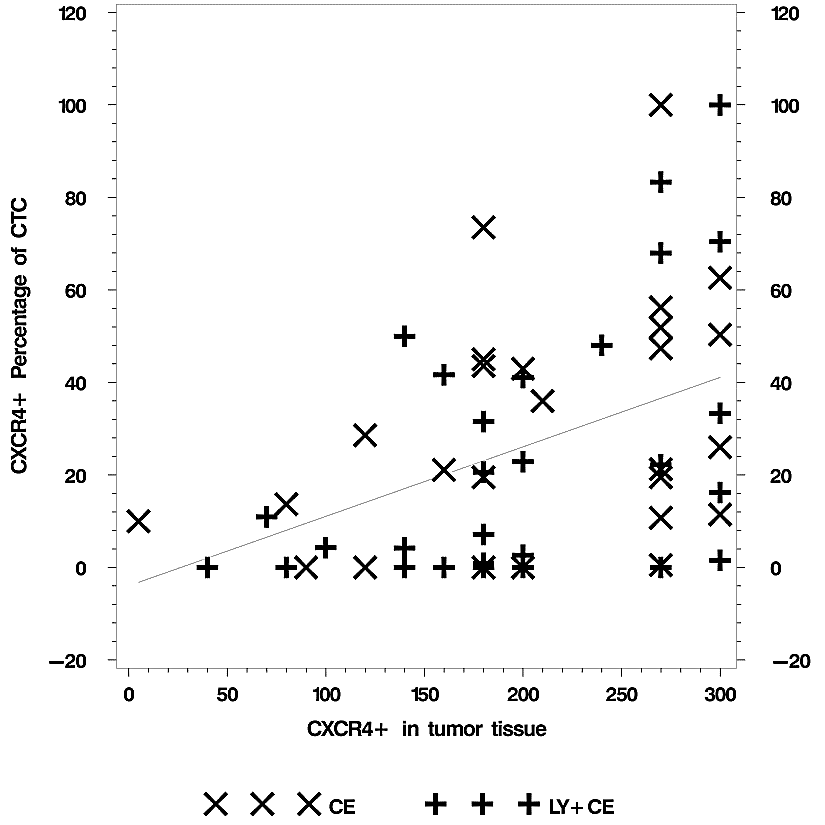


**b**


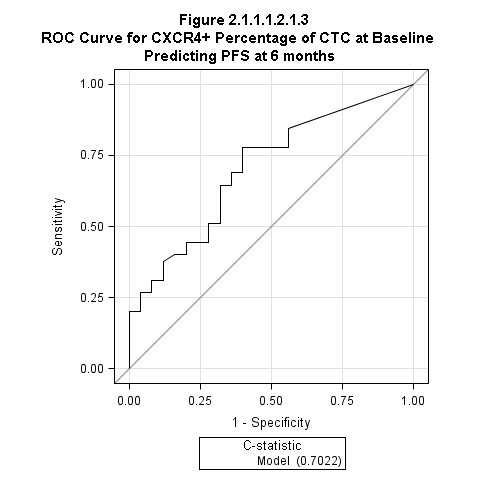


**c**


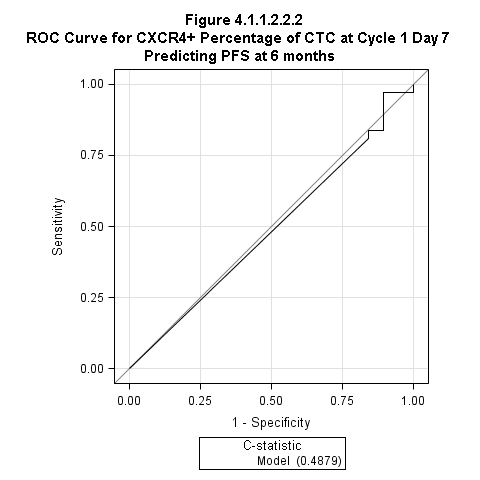


**d**


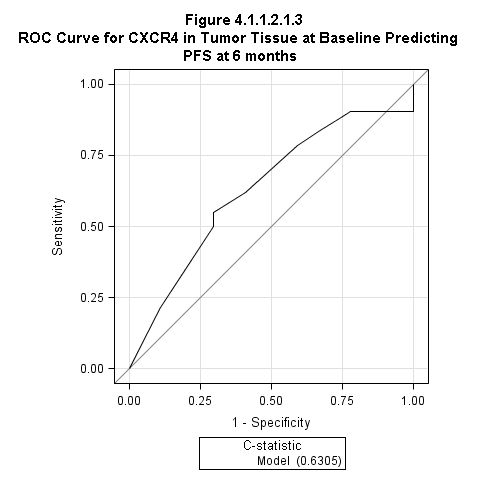


**e**


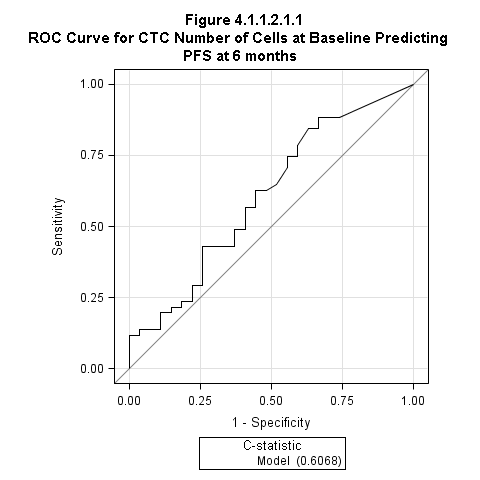


**f**


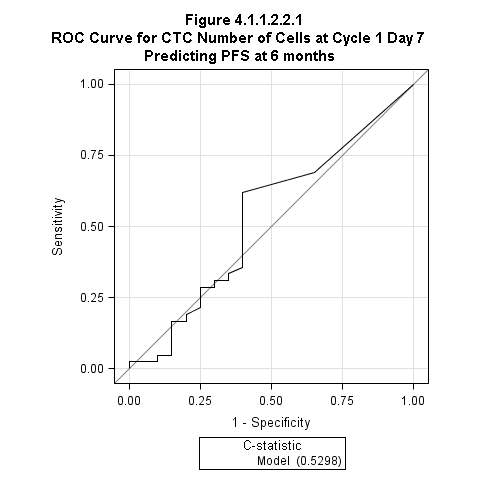


# Supplementary References

1. Paoletti C, Muniz MC, Thomas DG et al (2015) Development of circulating tumor cell-endocrine therapy index in patients with hormone receptor-positive breast cancer. Clin Cancer Res 21:2487-2498
